# COVID-19 and Mucormycosis Coinfection: How Challenging It Is

**DOI:** 10.1155/2022/8617212

**Published:** 2022-04-07

**Authors:** Niranjan Nayak, Erum Khan, Debadatta Panigrahi

**Affiliations:** ^1^Manipal College of Medical Sciences, Pokhara, Nepal; ^2^College of Medicine, Ajman University, Ajman, UAE

## Abstract

Recently, cases of rhinoorbital mucormycosis in people diagnosed with COVID-19 have been reported from India particularly. Diabetes mellitus though happens to be an independent risk factor both for severe COVID-19 and mucormycosis, administration of steroids is attributed as a precipitating factor for acquiring the comorbid condition. This opportunistic fungal infection is highly angioinvasive in nature because of which, clinical outcome of infection is invariably poor, especially with rhinocerebral or rhinoorbitocerebral variety of mucormycosis. However, effective management depends upon timely and accurate diagnosis and parenteral administration of amphotericin B. At the same time, judicious use of steroids is a key factor. In addition, glycemic control in those who are severely diabetic is strongly advocated. Exenteration of an eyeball may be indicated if cavernous sinus and intracranial spread are anticipated. Therefore, in order to facilitate faster healing and better penetration of antifungal drugs, surgical debridement of the paranasal sinus cavities and removal of dead tissue from the sinuses are recommended.

## 1. Introduction

Opportunistic bacterial and fungal infections are commonly encountered with COVID-19, caused by severe acute respiratory syndrome virus 2 (SARS-CoV-2) [1]. However, recently, an alarming number of cases of mucormycosis in people with COVID-19 have been reported worldwide, especially from India [2].

Mucormycoses are aggressive, angioinvasive, potentially fatal conditions caused by fungi of the *Mucorales* group, which affect patients with severe immunocompromised states. These immunosuppressed states tend to arise out of uncontrolled diabetes with diabetic ketoacidosis, long-term steroid therapy, hematological malignancies, solid organ transplant, and bone marrow transplant. From the abovementioned precipitating conditions, uncontrolled diabetes mellitus has been found to be a major factor in some parts of the world, including India, among the people manifesting severe invasive mucormycosis, in whom glycemic control was noted to be quite inadequate [3, 4].

Before diving the research details of COVID-19 and its alliance, if any, with mucormycosis, it is important to tend to a reoccurring misconception. In this regard, a bottom-line truth should be mentioned. Naming the fungi causing mucormycosis as “black fungi” is a misnomer, though the term “black fungi” is applicable to a group of fungi called dematiaceous fungi, which have the inherent property of producing black pigments. However, the dark brown lesions seen in mucormycosis, especially in the nose, paranasal sinuses, and hard palate, may be the reason for these fungi being named as “black fungi” by certain sectors.

## 2. Materials and Methods

### 2.1. Data Extraction

We examined PubMed using relevant keywords termed as ((zygomycosis OR mucormycosis OR *Mucorales*) AND (COVID-19 OR coronavirus OR SARS-CoV-2 OR pandemic)) to aid in finding qualitative and quantitative data. Following this procedure, analysis of literature published within last three years was executed, including the titles, abstracts, and results, aiding in narrowing all the analyses to selected studies for full text analysis according to their eligibility criteria.

### 2.2. Eligibility Criteria

Our full text analysis criteria included all human-based studies on COVID-19, mucormycosis, and mucor COVID-19 coinfection; although, in spite of this criterion, we chose to exclude any review articles.

### 2.3. Outcomes of Interest

The primary outcomes included COVID-19 and mucormycosis coinfection and its clinical parameters in patients. The therapeutic challenges faced by the otorhinolaryngologist, ophthalmologist, and physician were also examined to be included in the outcome analysis.

### 2.4. Quality Assessment and Data Collection

Author NN carried out the study selection and quality assessment. Authors NN, EK, and DP independently performed the data extraction, combining their individual results to reach a general conclusion.

### 2.5. Inclusion and Exclusion Criteria

We aimed to include case reports, case series, and observational studies describing the clinical features, pathogenesis, therapeutic modalities, and outcomes of mucormycosis in COVID-19 patients. Collectively, we found it unproductive to include case reports without details of laboratory findings and clinical features and pathogenesis.

Important analyses of risk factors, clinical presentations, diagnostic approaches, treatment modalities, and clinical outcomes were executed as well.

### 2.6. Study Characteristics

The search using the appropriate terms mentioned in the data extraction methods yielded 670 articles out of which 58 were relevant and fulfilled the eligibility criteria. We then further narrowed it down to only 19 for the discussion according to the homogeneity of these articles with ours, excluding the other 39. For further references, we provided pertinent information from the CDC/WHO. For our discussion on clinical presentations, treatment guidelines, and therapeutic challenges, we referred to some of the high-quality meta-analyses by the International Society for Human and Animal Mycology (ISHAM) and the European Confederation of Medical Mycology (ECMM).

## 3. Discussion

### 3.1. Mucormycosis: Clinical Entities

Mucormycosis is a severe life-threatening disease, which clinically manifests in the following forms: rhinocerebral mucormycosis or rhinoorbital cerebral mucormycosis, which often terminates fatally. Several varieties of mucormycosis can be clinically challenging, including mucormycosis of the lungs, gastrointestinal tract, disseminated mucormycosis which occurs in severely immunosuppressed individuals, and cutaneous mucormycosis which occurs after trauma and in contact with soil inhabited by *Mucorales* molds. Cutaneous mucormycosis may also be commonly found among burn care patients. Whereas rhinocerebral mucormycosis has frequently been observed in association with uncontrolled diabetes and diabetic ketoacidosis [5], pulmonary involvement has quite often been seen in patients having neutropenia, bone marrow and organ transplants, and hematological malignancies. On the other hand, gastrointestinal mucormycosis occurs usually in malnourished individuals [2]. Rhinocerebral or rhinoorbital cerebral variety with extensive involvement of the paranasal sinuses is, however, the commonest category which is dealt with by the otorhinolaryngologist and the ophthalmologist.

### 3.2. Mucormycosis and COVID-19: Clinician's Cognizance

In the recent past, COVID-19-associated pulmonary aspergillosis has been the central focus of attention in the literature so far as the COVID-19-associated secondary infections are concerned [6, 7]. Other fungal superinfections such as those due to *Candida* [8], rare mold infections like fusariosis [9], and COVID-19-associated mucormycosis [10, 11] are likely to be underreported. Hence, the purpose of this review was to highlight on COVID-19-associated mucormycosis, emphasizing particularly on the diagnostic and therapeutic challenges and effective management strategies.

Amongst all the risk factors predisposing COVID-19 patients to mucormycosis enumerated above, use of immunosuppressive agents is even more important because of the strong association of rhinocerebral mucormycosis with the abrogated immune status in the host, which is evidence based [12]. However, there are limited clues to find any causal relationship between the *Mucorales* group of fungi and COVID-19, in the inception of a superadded opportunistic fungal infection.

Notwithstanding the above, it was documented from the recent reports of emergence of mucormycosis among COVID-19 patients that many clinicians preferred administering steroids to compromise the magnitude of inflammation in the host due to COVID-19 virus [13]. Thus, it is appropriate to mention here that prescription of steroids should only be advocated, whenever patient is hypoxic with a substantial decline in oxygen saturation. As long as the status of oxygen saturation is within normal limits and the patient is having no other symptoms, except fever, dry cough, malaise, and mild joint pain, it is unnecessary to go with steroid treatment.

Even though, dexamethasone proved beneficial in certain groups of hospitalized COVID-19 patients; it can, on the other hand, potentiate the risk of invasive mold infections [14]. Additionally, corticosteroids can induce hyperglycemia which could synergize with the base line hyperglycemia due to undiagnosed or uncontrolled diabetes [15]. It was also documented that patients with severe diabetes and hyperglycemia often exhibited a state of inflammatory process that included constant recruitment and activation of macrophages and neutrophils secreting abundant amount of proinflammatory cytokines and generating persistent inflammation [16].

Hence, first, in the event of any indication for steroid therapy, the drug should always be prescribed in the recommended dosage, i.e., 6 mg/kg bodyweight/day of dexamethasone for a maximum period of 7–10 days [12]. Neither the dose nor the duration of therapy should be compromised.

Second, estimation of serum ferritin levels from time to time is essential, and elevated serum ferritin would certainly raise high index of suspicion of severe comorbidity with *Mucorales*. It must be mentioned here that organisms belonging to the order *Mucorales*, i.e., *Mucor, Rhizopus, Absidia, Cunninghamella, Rhizomucor*, and *Apophysomyces* have high affinity surface molecular receptors which could absorb ferritin from the host microenvironment [17], thus triggering the survival rate of the organisms.

Third, the key element to be reiterated is that majority of the COVID-19 patients come back with signs and symptoms of mucormycosis in about 14–18 days after getting discharged from the hospital, as proclaimed by several reports including those from India [15, 18]. This, therefore, becomes obituary on the part of the treating physician to counsel the patient on discharge, about the mild and common features of mucormycosis at its initial phase, such as facial pain and facial swelling, headache, fever, nasal blockage, and brownish nasal discharge, so that the patient may seek immediate medical attention. Once the patient comes for medical advice, with these typical commencing features, it is advisable to perform a paranasal sinus endoscopy and collect a suitable biopsied material from the snus cavity and send the sample to the microbiology laboratory for the direct microscopy and culture. Any well-equipped clinical laboratory should be successful in being able to provide the direct microscopy report by an hour's time, licensing the initiation of antifungal therapy.

The second most common form of infection due to the *Mucorales* seen in COVID-19 patients is pulmonary mucormycosis [19]. In such cases, there are many clinical and technical problems confronted with by a pulmonologist in diagnosing the case due to the following reasons. Primarily, both the pathogens, i.e., COVID-19 virus and the fungus, affect the same anatomical site i.e., the lung tissue. Hence, there are a number of clinical features which are common to both, such as fever, cough, pleuritic chest pain, and shortness of breath. Radiologically, one may find bilateral multilobular ground glass opacities with or without features of consolidation. In addition to these typical computerized tomographic findings, there may be some atypical ones in the form of pulmonary cysts, pleural effusion, hilar lymphadenopathy, pulmonary nodules, cavitation, and even pneumothorax. The aforementioned typical imaging features may be either due to COVID-19 per se or may be due to superimposed mucormycosis [20]. Over and above, pulmonary aspergillosis, too, can mimic mucormycosis to a large extent so far as the above radiological and clinical presentations are concerned [19].

To delineate further, diagnosis of COVID-19-associated mucormycosis, especially of the pulmonary variety, is truly challenging because the clinical and radiological features are very nonspecific, and there could be an overlap of these features in either of the conditions [10]. Detailed guidelines illustrate how to diagnose mucormycosis [10] and when to suspect COVID-19-associated mucormycosis [21].

Another example of challenging presentation in COVID-19 patients is acute respiratory distress syndrome (ARDS), which is invariably mistaken for other angioinvasive infection like pulmonary aspergillosis. Second, radiological features of pulmonary mucormycosis, i.e., the reversed halo sign could also be a prominent feature of the lung pathology in COVID-19 itself [22, 23]. Cavitary lung lesions though regarded as more specific than reversed halo sign in COVID-19-associated mold infections, are frequently observed both in COVID-19 aspergillosis coinfection as well as in COVID-19 mucormycosis coinfection [6].

The situation seems to have grown more intriguing in the absence of suitable biomarkers for mucor infections and unavailability of PCR assay in many laboratories in low and middle-income countries [24]. Therefore, it is needless to emphasize that accurate diagnosis of COVID-19 mucormycosis coinfection is still problematic, as the mainstay in the diagnosis remains with conventional culture and histopathological examination of biopsied tissue, despite the low sensitivity of both these investigations [24].

Last is the tough situation faced by the laboratory physician in getting the appropriate and adequate lower respiratory specimen like sputum or bronchoalveolar lavage fluid as majority of the patients are moribund, admitted to ICUs and are on indwelling devices.

### 3.3. Management of Cases and Therapeutic Challenges

Therefore, in context to the clinician's cognizance and in the setting of impracticability for a confirmatory diagnosis, it will be relevant to start antifungal drugs which are effective against *Aspergillus* as well as *Mucor, Rhizopus*, and other related fungi. Thus, prime management of patients with COVID-19 and mucormycosis coinfection would encompass the combined efforts propounded by the physician, the otorhinolaryngologist, and the ophthalmologist. The first and paramount step in this regard is glycemic control and omitting or reducing the dosage of steroids [13]. Second, surgical removal of necrotic material from inside the paranasal sinuses, and turbinates is nonetheless very important, keeping in view the acceleration of rapid healing process, as well as better drug penetration. Quite often, patients with rhinosinoorbital mucormycosis present with profuse swelling around the periorbital area, with proptosis, due to intense inflammation of the infraorbital tissue behind the eyeball [25]. This may lead to pan uveitis, and later, the infection from the orbit may extend in a retrograde manner via thrombotic emboli to the cavernous sinus and eventually to the brain [25, 26]. Most members of the *Mucorales* are seriously angioinvasive which may lead to finding an indurated eschar in the hard palate because of palatal perforation. These complications arising out of orbital cellulitis are invariably fatal. Consequently, to avoid intracranial spread and in order to save the life of the patient, it is highly recommended to exenterate the eyeball. Life is preferred, compromising the vision.

Apart from the surgical interventions, medical management of the cases is equally important. Administration of antifungal drugs is as important as surgical removal of dead tissue or organ. The most preferred antifungal agent which would take care of both *Mucorales* and *Aspergilli* is liposomal amphotericin B in a dosage of 5–10 mg/kg/day intravenously [27]. Such high dose can be administered safely as this formulation of amphotericin B is devoid of any nephrotoxicity or hepatotoxicity, unlike amphotericin deoxycholate. However, only disadvantage is that it is an expensive drug, and sometimes, it may be beyond the affordable reach of many patients in developing countries.

Amphotericin B deoxycholate, on the other hand, is cheap. It is given in a lower dosage, i.e., 1–1.5 mg/kg/day by slow intravenous (IV) infusion along with 5% dextrose solution [27]. Constant monitoring of renal parameters is important, while the patient is on amphotericin B deoxycholate. After clinical improvement, IV therapy is discontinued and the patient is put on oral antifungal agents like isavuconazole for a period of other 3-4 months. It may be mentioned here that drugs such as fluconazole, voriconazole, or echinocandins are ineffective against mucormycosis.

## 4. Conclusion

Mucormycosis and COVID-19 coinfection can be life threatening if a patient of COVID-19 having underlying diabetes develops rhinocerebral or rhinosinoorbitocerebral pathology. Yet, pulmonary mucormycosis occurring in a COVID-19 patient can be seen to be quite challenging both towards clinical and laboratory aspects of diagnoses. The complex interplay between mucor and COVID-19 virus is still not well understood. In conclusion, a multidisciplinary approach involving clinical microbiologist, otorhinolaryngologist, ophthalmologist, and physician is needed for the management of cases to be the most effective.
